# Interaction between multi-walled carbon nanotubes and propranolol

**DOI:** 10.1038/s41598-020-66933-7

**Published:** 2020-06-24

**Authors:** Wenjie Nie, Yani Li, Leyuan Chen, Zhicheng Zhao, Xin Zuo, Dongdong Wang, Lei Zhao, Xinyue Feng

**Affiliations:** 1 College of Geology and Environment, Xi’an University of Science and Technology, Xi’an, 710054 China; 2 Shaanxi Provincial Key Laboratory of Geological Support for Coal Green Exploitation, Xi’an, 710054 China

**Keywords:** Environmental sciences, Environmental chemistry

## Abstract

Carbon nanotubes could accumulate in organism and have a negative impact on the structure and function of the ecosystem when they were discharged into environment. Furthermore, it will affect the migration and fate of pollutants in the water body. The study is mainly to explore the adsorption behavior and mechanism of beta-blocker on multi-walled carbon nanotubes (MWCNTs). Propranolol (PRO) was selected as the representative of beta-blocker. The effects of different environmental factors such as pH, ionic strength and humic acid (HA) on the adsorption process were investigated. The adsorption results were characterized by Zeta potential. At the same time, the effects of different types of drugs on the adsorption process were explored and the possible adsorption mechanisms were analyzed. The experimental results showed that the adsorption behavior was significantly different under different pH conditions. π-π EDA interaction, hydrophobic interaction and hydrogen bonding were speculated to be the main adsorption mechanisms for PRO adsorption on MWCNTs.

## Introduction

In recent years, with the large-scale industrialization of nanotechnology, carbon nanotubes (CNTs), as a new type of nanomaterial with large specific surface area and high surface energy, are widely used in biomedicine, electro-optics, smart sensors and catalytic degradation^1–5^. However, CNTs are often discharged into the body of water without any treatment during production, application, and disposal, which may damage the balance of the ecosystem and seriously affect the normal ecosystem^6–8^. At present, many studies have shown that CNTs can adversely affect many organisms. CNTs can cause delayed incubation of zebrafish embryos, which was ascribed to an interaction between a large number of functional groups on the surface of CNTs and the eggshell’s hatching enzymes, inhibiting their activity and slowing the dissolution of the egg membrane^9^. The normal physiological functions of the cell membrane were influenced due to the damage of cell integrity and the organelles because CNTs will directly enter the cell through the cell membrane^10–12^. Furthermore, the strong adsorption capacity of CNTs will also change behaviors such as migration, transformation, and fate of other pollutants in the environment^13–15^. At the same time, CNTs adsorbing environmental pollutants will form various types of composite pollutants, further causing greater harm to the environment. Therefore, more attention should be paid to the adsorption behavior of CNTs.

Based on the interaction between CNTs and contaminants including heavy metals, antibiotics, and pesticides, some studies have pointed out that CNTs are important carriers or adsorbents to affect the migration and transformation of pollutants. The interaction mechanisms between CNTs and heavy metals are very complicated and may attributable to electrostatic attraction, sorption-precipitation and chemical interaction between the metal ions and the surface functional groups of CNTs^16,17^. The adsorption of antibiotics on CNTs was affected by the properties of CNTs such as specific surface area, oxygen content and adsorption sites, and adsorption heterogeneity and hysteresis are two characteristics of the interaction between antibiotics and CNTs^18,19^. CNTs are also used as excellent adsorbents for the removal of pesticides such as diuron and dichlorophenylhydrazine^20^. Beta-blockers are widely used to treat cardiovascular diseases, which that existed as parent compound cannot be completely removed by a sewage treatment plant, resulting in residues in natural waters, which have adversely affect organisms even at low concentrations^21–24^. Now a study had shown that atenolol, one of beta-blockers, could interact with CNTs in water^25^. PRO is one of the top selling beta-blockers. The current researches focus on the removal of PRO^26–28^, and the adsorption behavior of PRO on CNTs has not been systematically studied.

CNTs are classified into single-walled carbon nanotubes (SWCNTs) and multi-walled carbon nanotubes (MWCNTs). MWCNTs were selected as the research object because MWCNTs are easy to form depressions between layers when they are formed compared with SWCNTs. The walls of MWCNTs are usually covered with small hole-like defects and the surface performs more actively. The aims of this work were to investigate the adsorption behavior of PRO onto MWCNTs and to explore the adsorption mechanism. The effects of different influencing factors on the adsorption capacity were studied systematically. Reasonable conclusions were drawn according to the experiment results.

## Materials and Methods

### Materials

MWCNTs were supplied by the Shenzhen Tuling Evolution Technology Co., Ltd, China. The MWCNTs are about 15–30 μm long with an outer diameter of 3–15 nm. The BET surface area determined by N_2_ adsorption is about 260 m^2^/g and the ash content is less than 2.5 wt.%. Metoprolol tartrate (MTL) and carbamazepine (CBZ) were obtained from Aladdin Industrial Corporation (Shanghai, China). All chemicals used here were of analytical grade and used without purification.

### Adsorption experiments

The experiments were conducted according to reference^29^. PRO was adsorbate and MWCNTs was adsorbent. All batch experiments were carried out at 25 ± 2 °C. All adsorption experiments were performed in 50 mL centrifuge tubes containing 40 mL of certain PRO concentration and 0.01 g MWCNTs. Adsorption kinetic was conducted in a certain time range (5min-10h). Different concentrations of PRO (10–100 mg/L) were investigated in isotherm experiment. The environmental factors HA (5–200 mg/L), and Na^+^ and Ca^2+^ (0–250 mg/L) were considered to explore the further adsorption mechanism. Different concentration of CBZ and MTL were also added at varying concentration (5–25 mg/L) at varying pH (pH = 3–11.5) to study the effect of different types of pharmaceutical. The reaction system was shaken for 24 h to reach adsorption equilibrium except adsorption kinetic, pH was kept as 6.5 except pH experiment, and 25 mg/L was used as PRO concentration except isotherm experiment.

The quantities of adsorbed PRO were calculated by the difference of the initial and residual amounts of PRO in solution divided by the weight of the adsorbent (MWCNTs) after multiplying by solution volume. Duplicate samples were run for all of the experiments and only the average values were recorded. The methanol content in the solution was kept below 0.1% by volume to minimize the cosolvent effect. Blank samples were carried out for each experiment and did not indicate obvious degradation or loss during the whole experiment process.

### HPLC analysis

The solid and liquid on reaction system were separated by a filter membrane after the adsorption was completed. The PRO concentrations were measured by HPLC (Agilent 1200, USA) with a 4.6μm × 150 mm × 5 μm reverse phase XDB-C18 column and a UV detector. 40 μL PRO solution was injected into HPLC system with mobile phases contained 0.1% formic acid-acetonitrile and acetonitrile (70:30, v/v) at 1 mL/min flow rate. The analytical wavelength of PRO was set at 290 nm. The solvent system was cleaned and degassed before use.

### Characterization methods

Zeta potentials of MWCNTs were measured (Malvern, UK) to observe the surface charging of MWCNTs before and after adsorption. pH at the point of zero charge (pH_pzc_) was determined when zeta potential equaled zero.

## Results and discussion

### Adsorption kinetic

**Figure 1:**
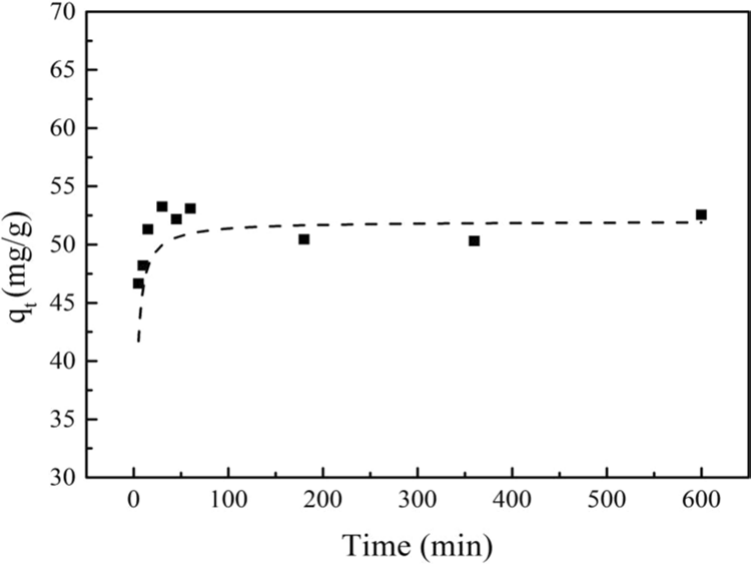
Adsorption kinetics of PRO on MWCNTs.

The adsorption equilibrium time of PRO on MWCNTs was determined by kinetic experiments. The relate curve between reaction time and adsorption capacity was depicted in Fig. 1. At the first 30 min, PRO adsorption on MWCNTs increased sharply with time increasing, then slowed down gradually, and finally maintained a platform after 60 min. It was learned that the adsorption sites were occupied quickly by PRO at the beginning of the adsorption process. As the surface sites of MWCNTs were gradually occupied, the adsorption equilibrium was finally reached.

Experimental data were fitted by the pseudo-second-order model (Eq. (1)).

1 $$\frac{t}{{q}_{t}}=\frac{1}{K{q}_{e}^{2}}+\frac{t}{{q}_{e}}$$

where *q*_*e*_ and *q*_*t*_ are the amount of PRO adsorbed onto MWCNTs at equilibrium and at time *t*, respectively; *K* is the constant of pseudo-second-order rate.

The kinetic curve was shown in Fig. 1. The theoretical data and the fitted parameters were shown in Table 1. It can be seen that the experimental data were consistent with the pseudo second-order kinetic model based on a high correlation coefficient (R^2^ = 0.9993). The *q*_*e*_ value calculated by the pseudo second-order kinetics was very close to experimental data. Therefore, the kinetic data of PRO adsorption on MWCNTs were more in line with the pseudo second-order kinetic model.

**Table 1:** Kinetic parameters for PRO adsorption on MWCNTs.

C_0_ (mg/L)	q_exp_ (mg/g)	Pseudo-second-order equation		
		K (g/(mg min))	q_cal_ (mg/g)	R^2^
50	52.54	0.0156	51.75	0.9993

### Adsorption isotherm

**Figure 2:**
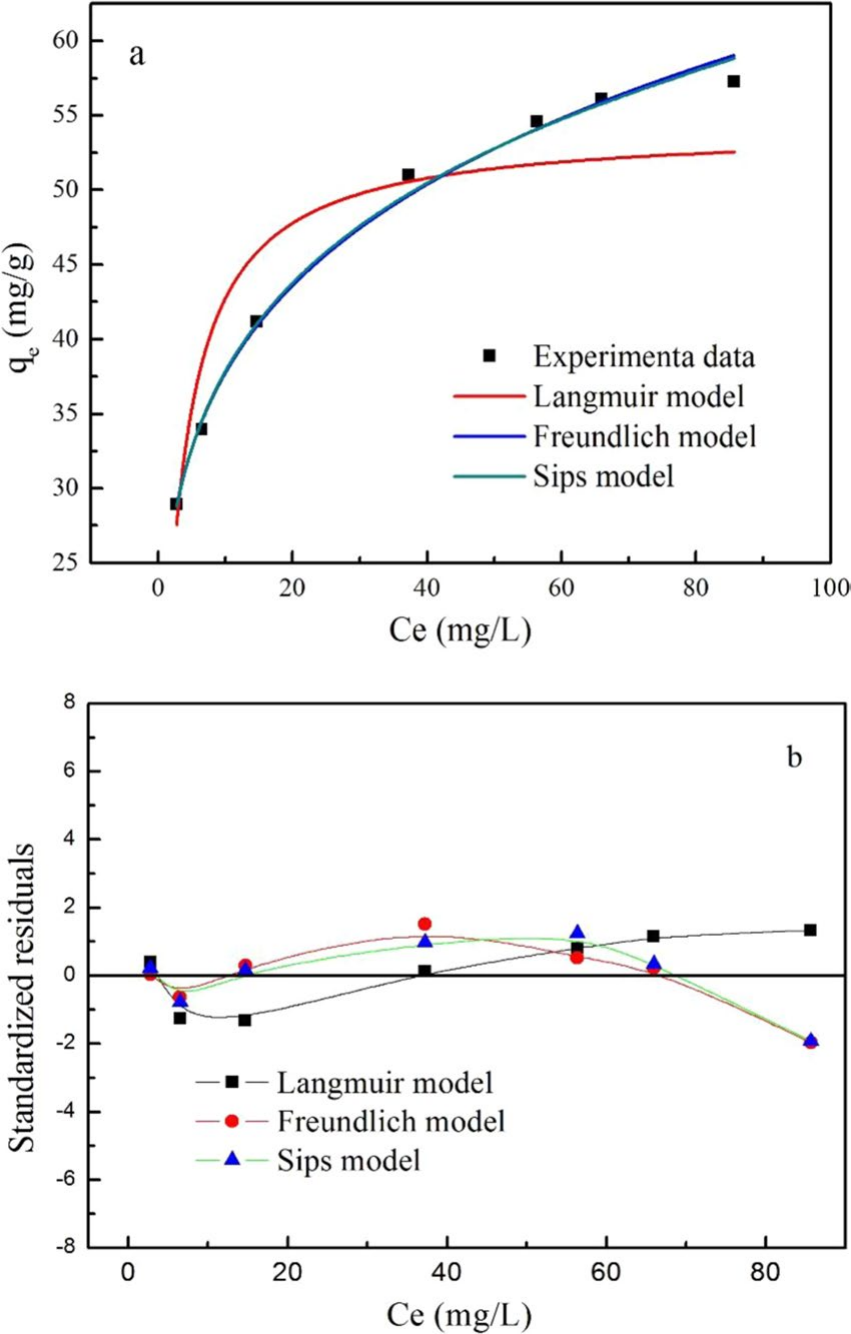
(**a**) Adsorption isotherm of PRO on MWCNTs. (**b**) Residual plot for data for Langmuir, Freundlich, and Sips models.

The trend of PRO adsorption capacity as a function of different PRO concentration was presented in Fig. 2(a). As shown, the adsorption capacity enhanced sharply with PRO concentration increasing at low values, then showed a slowly increase, and finally maintained as a constant. At a lower concentration, PRO molecules were easily attached to adsorption sites without competition among PRO molecules. Further, the PRO transportation from solvent to MWCNTs surface was promoted by the increase of PRO concentration. However, the adsorption sites on the surface of MWCNTs were stressed due to an increasing of the number of adsorbate molecules, resulting adsorption relaxation at high concentration.

Adsorption isotherm can provide more information about the adsorption mechanism of PRO on MWCNTs. The experiment data were simulated with Langmuir, Freundlich, and Sips models. The expression of the Langmuir (Eq. (2)), Freundlich equation (Eq. (3)), and Sips equation (Eq. (4)) were shown as followed.

2 $${q}_{e}=\frac{{q}_{m}{K}_{L}{C}_{e}}{1+{K}_{L}{C}_{e}}$$

3 $${q}_{e}={K}_{F}{{C}_{e}}^{\frac{1}{{n}_{1}}}$$

4 $${q}_{e}=\frac{{q}_{m}{(b{C}_{e})}^{\frac{1}{{n}_{2}}}}{1+{(b{C}_{e})}^{\frac{1}{{n}_{2}}}}$$

where *q*_*m*_ represents the maximum amounts of the adsorbed PRO per unit mass of MWCNTs. *Ce* is PRO concentration in solution at equilibrium. *q*_*e*_ donates the PRO equilibrium concentration on MWCNTs. *K*_*L*_ is Langmuir adsorption constant, which relates to the affinity and adsorption energy of the bonding sites. *K*_*F*_ is Freundlich constant also as known as a capacity factor associated with the adsorption capacity and the adsorption strength. 1*/n*_1_ is the heterogeneity factor. The value of 1/*n*_*1*_ is between 0–1, which characters the effect of concentration on the amount of adsorption. 1/*n*_2_ represents the heterogeneity of the sorbent. *b* is the median association constant^30^.

Figure 2(a) also displayed the fitted curves of Langmuir, Freundlich, and Sips. The relative parameters calculated from the two models for MWCNTs were listed in Table 2. The Langmuir model assumes that uniform adsorption sites are distributed on the surface of adsorbent and only a single layer adsorption will occur, which was inconsistent with the experimental data (*R*^2^ = 0.8828) and the Langmuir model was considered to be an unreasonable descriptor. The applicability of the Freundlich model (*R*^2^ = 0.9943) suggested that MWCNTs possessed different types of surface sites, which was reasonable for MWCNTs that contained gap, functional groups and groove region between bundles. n was much more than 1, reflecting the high adsorption nonlinearity and the favorable adsorption. Sips model is derived from Langmuir model and Freundlich model. The correlation coefficient of Sips was a bit higher than that of Freundlich, which suggested that the reaction process was consistent with single-layer adsorption at low PRO concentrations and multilayer adsorption at high PRO concentrations. Figure 2(b) was residual plot for data for Langmuir, Freundlich, and Sips models. The residual plots also clearly showed that the standardized residuals are more randomly distributed around zero for Sips, which suggested Sips fitted the experimental data well.

**Table 2:** Calculated parameters for Langmuir and Freundlich isotherms models for PRO adsorption on MWCNTs.

Langmuir equation					Freundlich equation				Sips equation			
*q*_*m*_ (mg/g)		*K*_*L*_ (L/mg)		*R*^2^	*K*_*F*_ (mg^1–1/n^•L^1/n^•g^−1^)		*n*_1_	*R*^2^	*q*_*m*_ (mg/g)	*b*	*n*_2_	*R*^2^
54.17	0.3731		0.8828		23.3620	4.8012		0.9943	380.22	−31.702	4.254	0.9955

### The effect of HA

**Figure 3:**
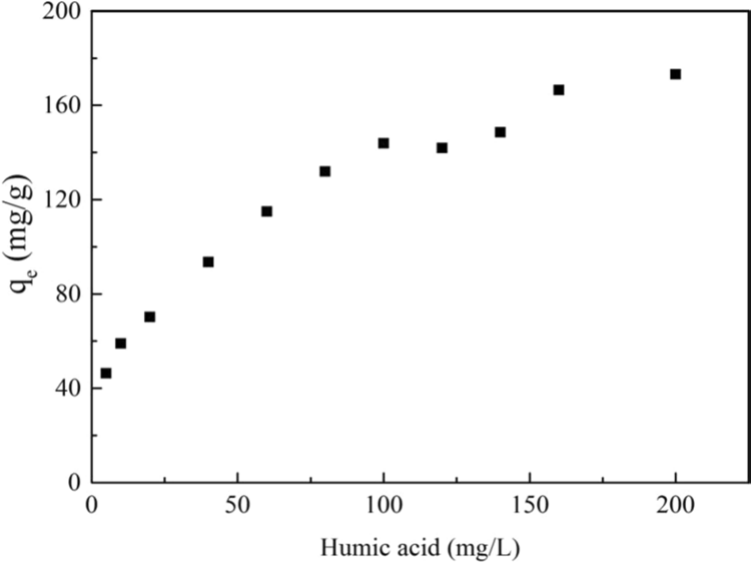
The effect of humic acid for the adsorption of PRO on MWCNTs.

HA, widely existed in aqueous solution, could interact with CNTs and organic contaminant^31^. Figure 3 exhibited the effect of HA on the PRO adsorption on MWCNTs. Obviously, the adsorption capacity of PRO on MWCNTs increased as the HA concentration increased. HA was adsorbed by MWCNTs that contained huge specific surface area and surface defects, which enhanced the hydrophilic, steric hindrance and electrostatic repulsion of MWCNTs. The hydrophobic sites on the surface of MWCNTs were occupied partly by HA, providing more available adsorption sites (eg. oxygen-containing functional groups, aromatic rings) for the adsorption of cation form PRO. Meanwhile, the positive charges on the surface of MWCNTs were neutralized by HA with the negative charges, weakening the electrostatic repulsion between cation form PRO and MWCNTs. Nevertheless, the enhancement was slowed down after reaching a certain concentration (HA > 100 mg/L), which may attribute to a saturated trend of HA adsorbed on MWCNTs.

### The effect of ionic strength

**Figure 4:**
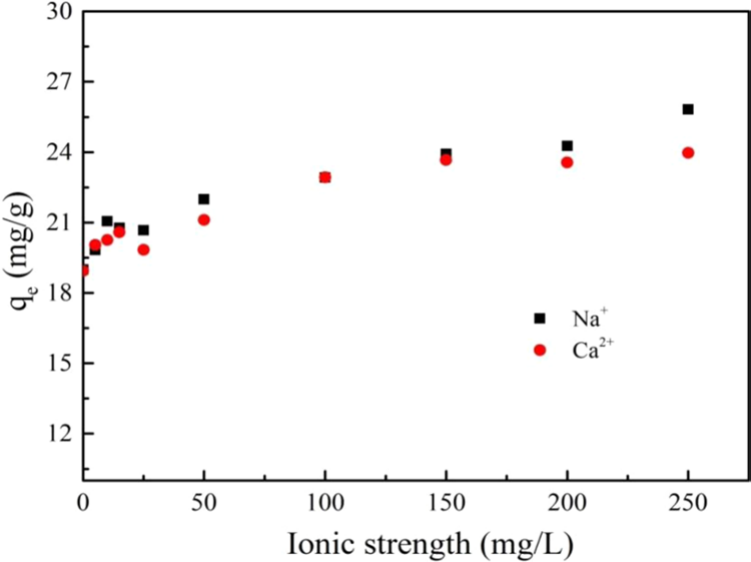
The effect of Na^+^ and Ca^2+^ for the adsorption of PRO on MWCNTs.

Ionic strength was an important factor controlling the adsorption process. The effect of Na^+^ and Ca^2+^ on the adsorption of PRO on MWCNTs was depicted in Fig. 4. In general, the ions of the two valence states promoted the adsorption of PRO on MWCNTs with the increase of ionic strength, but the promotion effect was not obvious. The introduction of Na^+^ and Ca^2+^ with strong hydration reduced the number of water molecules originally attracted around PRO. The hydrophilicity of the PRO was reduced and the hydrophobicity was relatively enhanced. PRO was more likely to be adsorbed on the surface of MWCNTs by hydrophobic interaction^32^. In addition, the increase in ionic strength led to the aggregation of PRO molecules, the adsorption capacity of PRO on MWCNTs increased. However, the promotion was so weak that the effect of ion valence on adsorption was negligible, indicating the high stability of interaction between MWCNTs and PRO in a certain range of salt concentration.

### The effect of pH

**Figure 5:**
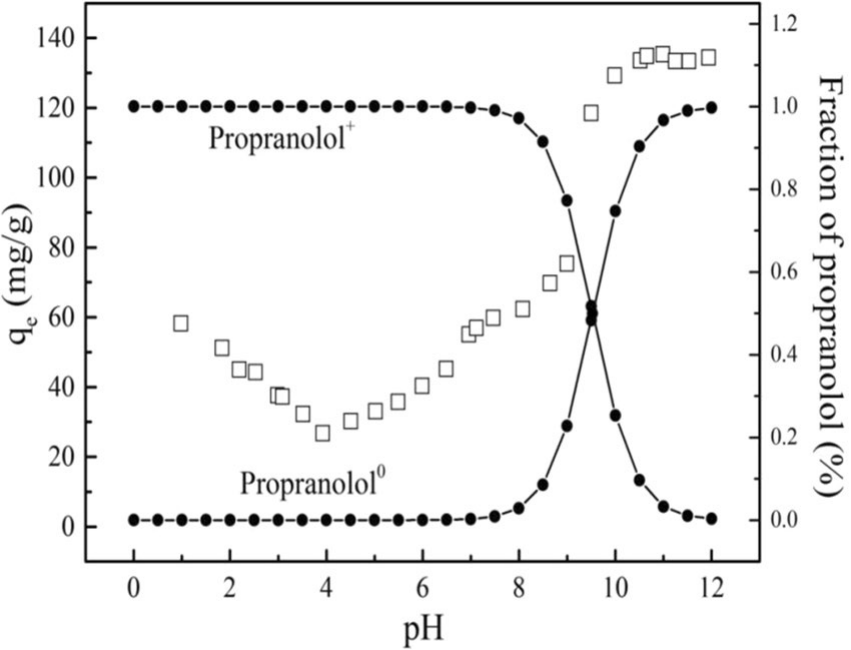
The effect of pH for PRO adsorption on MWCNTs at a given concentration and the fraction of PRO at different pH.

The surface properties of MWCNTs and the existing form of PRO are affected by the reaction system pH, resulting in changes in the adsorption characteristics. Figure 5 showed the adsorption capacity of PRO on MWCNTs and the fraction of PRO at different pH. It can be seen that when the pH was lower than 4, the adsorption capacity decreased as the pH increased. Conversely, when the pH was >4, the adsorption capacity showed an opposite tendency. When pH was >7, the solution pH seemed to have little effect on the adsorption of PRO.

**Figure 6:**
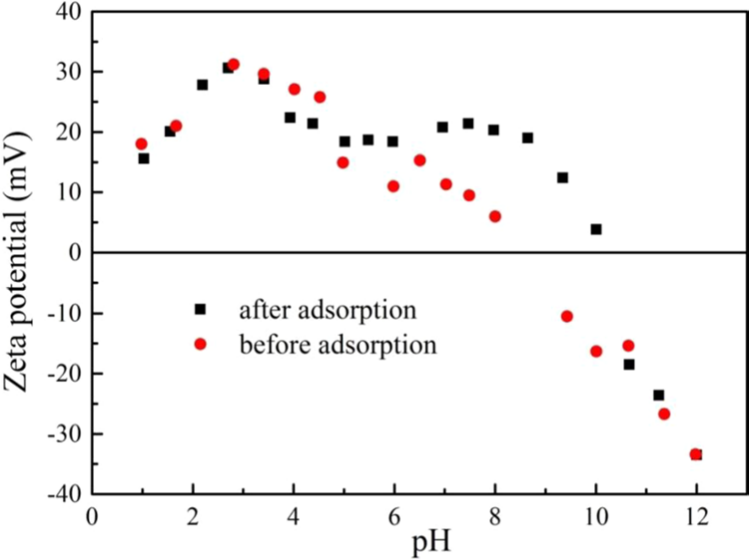
The changes of zeta potential before and after PRO adsorption on MWCNTs.

**Figure 7:**
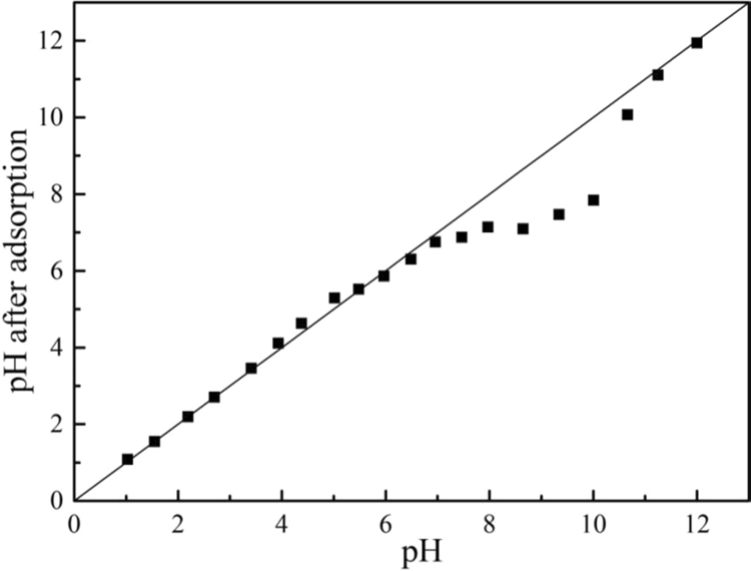
pH changes before and after PRO adsorption on MWCNTs.

The hydrophobic effect was one of the mechanisms by which MWCNTs adsorbed organic compounds due to the hydrophobic sites uniformly distributed at the outer surface of MWCNTs. At pH was lower than 3, the adsorption capacity decreased with pH increasing. The surface of MWCNTs was positively charged which increased with pH increasing (Fig. 6), meanwhile PRO was positively charged (pKa = 9.53), leading to a reduction adsorption due to electrostatic repulsion between them. When pH was >4, the adsorption capacity began to increase. The electrostatic repulsion was slightly weakened since the zeta potential of the MWCNTs was slightly decreased. At the same time, the hydrophilicity of PRO increased due to the presence of PRO in the form of a cation, resulting in the PRO ionized amino group possibly forming a cation-π interaction with MWCNTs by a similar π-π action. While, the H^+^ concentration in the system was higher, and the hydrogen bond acceptor of MWCNTs and the hydrogen bond donor of PRO were more likely to bind to H^+^, resulting in a decrease in hydrogen bonding between them. Therefore, the adsorption affinity of PRO was not strong low at lower pH. It can be seen from Figs. 6 and 7 that the zeta potential and pH before and after the reaction hardly change at pH < 7, indicating that the adsorption of PRO on MWCNTs was mainly dominated by hydrophobic interaction and cation-π interaction rather than static electricity at low pH.

At the pH range from 7 to 10, the adsorption capacity increased rapidly, especially in the vicinity of the pKa of PRO. The form of PRO gradually changed from the cationic form to the neutral molecular form. The increase in hydrophobicity enhanced the hydrophobic interaction between MWCNTs and PRO while the electrostatic repulsion between MWCNTs and PRO was weak, making PRO molecules more accessible to the surface of MWCNTs. PRO molecules with benzene ring structure can form π-π bonds with MWCNTs that each carbon atom had a π electron orbit around the surface perpendicular to the MWCNTs. The zeta potential in this range gradually decreased, and the zeta potential after adsorption was higher, which indicated that the adsorption of PRO led to an increase in surface potential of MWCNTs. Further, the pH after the reaction was smaller than the pH before the reaction, indicating that the H^+^ concentration in the reaction system was increased. It can be reasonably assumed that cation exchange may play a slight part in the adsorption process. At higher pH, the hydrogen bond donors on the surface of MWCNTs were ionized, while PRO was gradually deprotonated in the form of neutral molecules. Therefore, the hydrogen bond donor of PRO will interact with the hydrogen bond acceptor or π-donor of MWCNTs to promote adsorption. Interestingly, when the pH was close to the isoelectric point of MWCNTs, the electrostatic repulsion between the independent bundles will be weakened, resulting in the carbon nanotubes being more likely to aggregate. However, the increased adsorption capacity suggested that the aggregation of MWCNTs caused by pH changes had no significant effect on the adsorption process. The adsorption of PRO in MWCNTs mostly occurred on the outer surface of MWCNTs rather than in the trench space.

When pH was above 10, the adsorption capacity of PRO was kept at a high value, which was hardly affected by pH changes. PRO existed almost entirely in the form of neutral molecules, meaning the strongest hydrophobic. Hydrophobic interaction, hydrogen bonding and π-π action had played important roles in the adsorption process of MWCNTs.

### The effect of different types pharmaceuticals and adsorption mechanism

More and more pharmaceutical residues will seep into nature system, which affected the migration of organic contaminant. MTL and CBZ were chosen for further exploring the adsorption mechanism of PRO onto MWCNTs.

**Figure 8:**
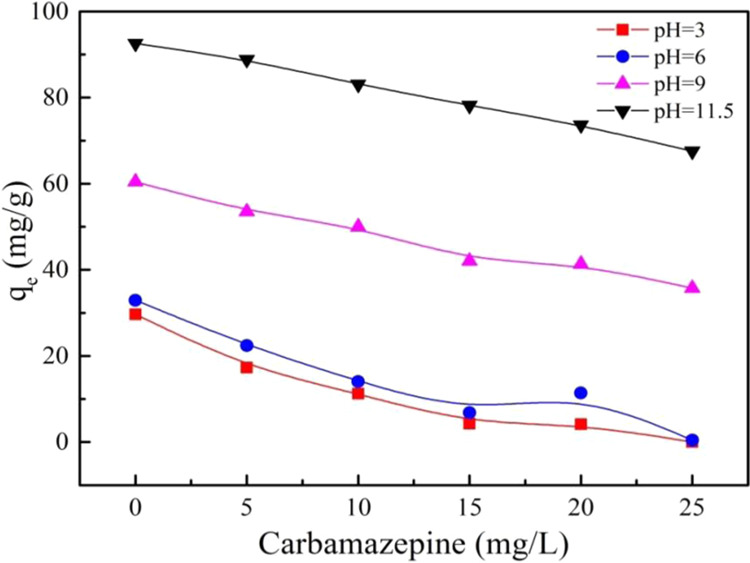
The different concentration effect of carbamazepine for PRO adsorption on MWCNTs different pH.

As shown in Fig. 8, the adsorption of PRO on MWCNTs was inhibited with the coexistence of CBZ at varying pH and concentration. The more CBZ molecules were, the stronger inhibition was, which suggested that CBZ competed similar adsorption sites on MWCNTs with PRO. The adsorption of CBZ on MWCNTs was exhibited in Fig. S1. Uptrend curves indicated that adsorption capacity enhanced with CBZ concentration increasing. π-π EDA interaction between the planar conjugated π-electron systems of CBZ and the graphene surface of MWCNTs was mainly attributed to the adsorption behavior of CBZ on MWCNTs, which led to competitive adsorption with PRO that had two benzene rings in PRO structure, attaching with MWCNTs by π-π interaction. However, the inhibition of PRO adsorption was limited at a certain extent due to the fact that the two pharmaceutical molecules do not share the exact same sites. It can be noted that the CBZ adsorption affinity on MWCNTs was not affected by pH of reaction system but PRO was. CBZ existed in a non-ionic form at the test range, but PRO, ionizable organic compounds, was existed as cation when pH <pKa. A low PRO adsorption capacity was obtained due to the electrostatic repulsion between PRO and MWCNTs, while no electrostatic interaction between CBZ and MWCNTs. Meanwhile, the cation-π bonds form between the protonated secondary amines of PRO and the surface of MWCNTs may attribute to a certain adsorption of PRO. Furthermore, covalent bonds that may form between amide group on the structure of CBZ and the surface of MWCNTs were responsible for the liner CBZ adsorption. Other than this, Lerman *et al*. proposed that CBZ may interact with sites other than external surfaces and groove regions of CNTs^33^.

**Figure 9:**
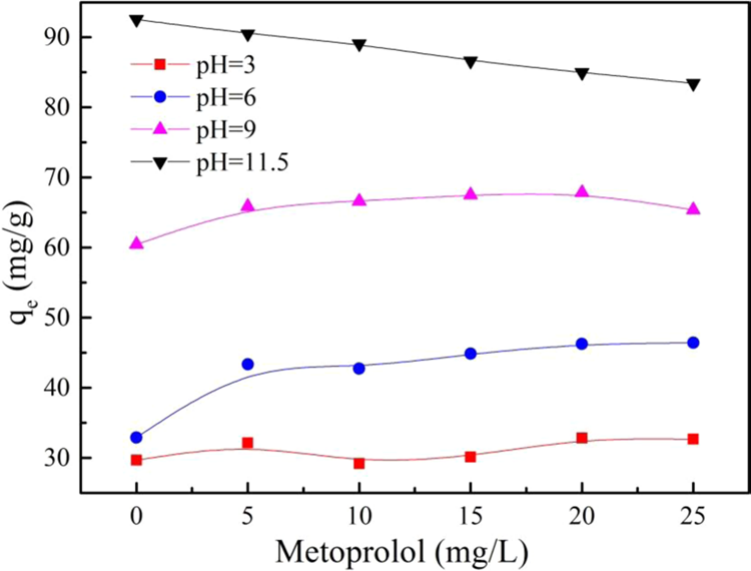
The different concentration effect of metoprolol for PRO adsorption on MWCNTs different pH.

MTL was ionizable organic compounds of cation form as same as PRO with pKa = 9.2 or 9.6^34^. The effect of MTL on PRO adsorption was shown in Fig. 9. It can be seen that MTL had little effect on PRO adsorption with an increasing concentration of MTL at pH = 3, 6, and 9, while a slight decrease of PRO adsorption capacity at pH = 11.5. The adsorption of MTL on MWCNTs was depicted in Fig. S2, which presented that the adsorption capacity of MTL increased with an increasing concentration, particularly at pH = 11.5. MTL has the same structure and properties with PRO, two of them are the members of beta-blockers family. Competitive adsorption should occur between PRO and MTL, which suppressed PRO adsorption affinity. However, the experimental data indicated a subtle inhibition. It was reasonable to speculate that the phenomenon was caused by the different structures of MTL had a benzene ring and PRO had two benzene rings. Compared with MTL, it was easier for PRO to interact with MWCNTs, which was consistent with the experimental data. The coexistence of MTL had hardly any influence on π-π EDA between PRO and MWCNTs. A slight inhibition may occur since MTL competed with PRO for hydrogen bond.

In general, the main adsorption mechanisms of PRO onto MWCNTs were ascribed to hydrogen bonding between the oxygen-containing functional groups on the surface of MWCNTs and N and O elements in PRO molecules, the hydrophobic interaction between the graphene-like surface of MWCNTs and the hydrophobic structure of PRO, and π-π interaction that electron-donor transfer mechanism formed between benzene rings structure of MWCNTs and PRO.

## Conclusions

In this study, the adsorption behavior of PRO on MWCNTs was investigated by batch experiments. The effects of pH, ionic strength and HA on the adsorption process were systematically investigated. The effects of two different type drugs on PRO adsorption were compared and the adsorption mechanisms of PRO on MWCNTs were analyzed. The ionic strength has little effect on the PRO adsorption process. The presence of HA promoted the adsorption of PRO on MWCNTs. The adsorption behavior of PRO at different pH was different. Combined with the experimental results of the effects of three drugs on PRO adsorption, the adsorption mechanism of PRO on MWCNTs can be mainly attributed to π-π EDA interaction, hydrophobic interaction and hydrogen bonding.

## Supplementary information

Supplementary information.
