# Supplementary material for: Interaction between multi-walled carbon nanotubes and propranolol
Source: Sci Rep. 2020 Jun 24;10:10259. doi: 10.1038/s41598-020-66933-7 (PMC7314780; doi:10.1038/s41598-020-66933-7)

Interaction between multi-walled carbon nanotubes and propranolol

Wenjie Nie^1, 2*^, Yani Li^1, 2^, Leyuan Chen^1^, ZhiCheng Zhao^1^, Xin Zuo^1^, Dongdong Wang^1^, Lei Zhao^1^, Xinyue Feng ^1^

^1^ College of Geology and Environment, Xi'an University of Science and Technology, Xi'an 710054, China;

^2^ Shaanxi Provincial Key Laboratory of Geological Support for Coal Green Exploitation, Xi'an 710054, China.

***** Correspondence: 76977485@qq.com

**Figure S1.** The adsorption of carbamazepine on MWCNTs


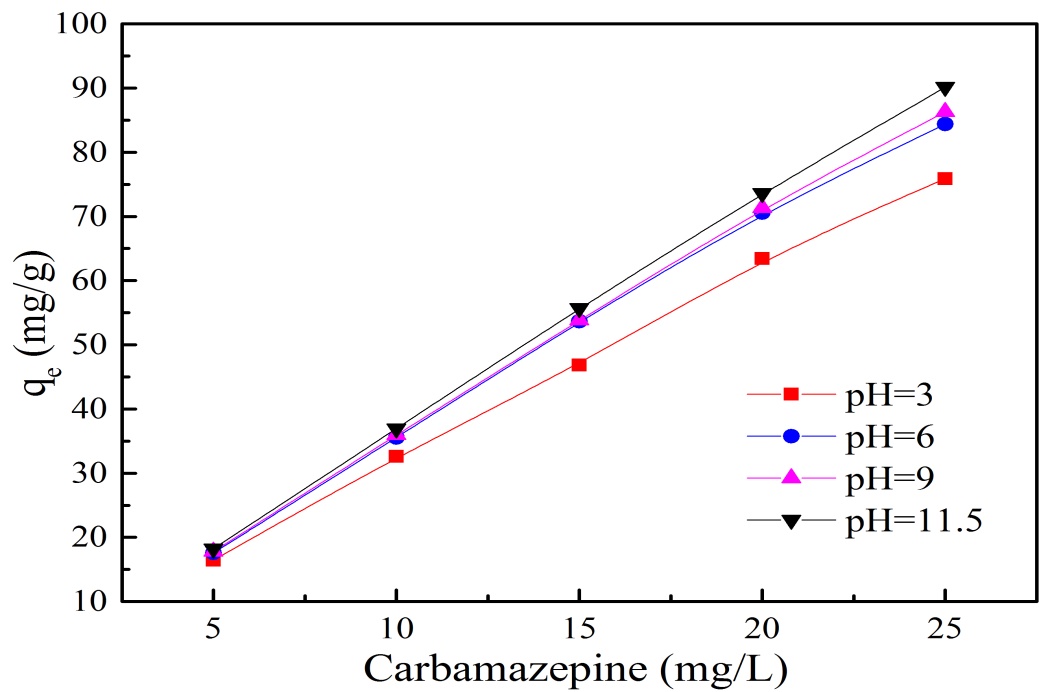


**Figure S2.** The adsorption of metoprolol on MWCNTs


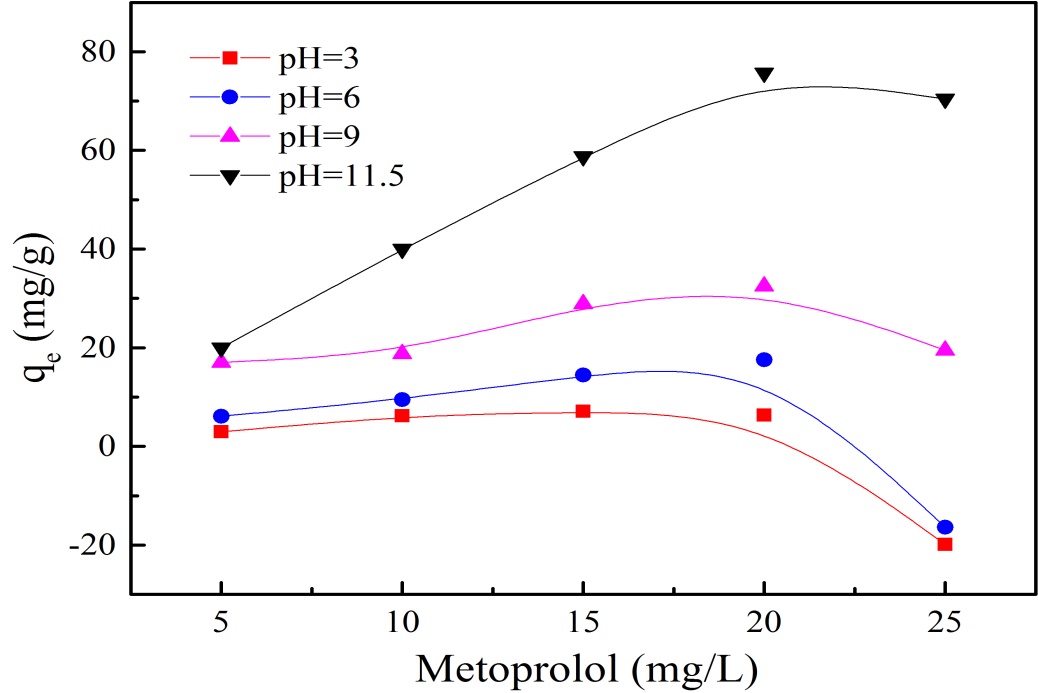

Supplement: Supplementary file 1 — Supplementary information. [file 41598_2020_66933_MOESM1_ESM.docx]
